# Evaluation of nutritional value and microbiological safety in commercial dog food

**DOI:** 10.1007/s11259-021-09791-6

**Published:** 2021-04-26

**Authors:** Katarzyna Kazimierska, Wioletta Biel, Robert Witkowicz, Jolanta Karakulska, Xymena Stachurska

**Affiliations:** 1 Department of Monogastric Animal Sciences, Division of Animal Nutrition and Food, West Pomeranian University of Technology in Szczecin, 29 Klemensa Janickiego, 71270 Szczecin, Poland; 2 Department of Agroecology and Crop Production, University of Agriculture in Krakow, 21 Mickiewicza, 31120 Krakow, Poland; 3 Department of Microbiology and Biotechnology, West Pomeranian University of Technology in Szczecin, 45 Al. Piastow, 70311 Szczecin, Poland

**Keywords:** Bacteria, Extruded food, Comparison analysis, Proximate composition, Energy value, Molds

## Abstract

In addition to properly balancing nutritional value in accordance with the needs of a dog, estimating the microbiological quality of dog food is crucial in providing healthy and safe foods. The aim of this study was to examine the quality of dry food for adult dogs, with particular reference to: (1) evaluating the nutritional value and compliance with nutritional guidelines for dogs, (2) comparing the nutritional value of dog foods, with particular emphasis on the division into cereal and cereal-free foods, and (3) evaluating their microbiological safety. All thirty-six evaluated dry dog foods met the minimum European Pet Food Industry FEDIAF requirement for total protein and fat content. The total aerobic microbial count in the analyzed dry dog foods ranged from 2.7 × 10^2^ to above 3.0 × 10^7^ cfu/g. In five (14%) dog foods the presence of staphylococci was detected; however, coagulase positive Staphylococcus (CPS) was not found. Mold presence was reported in one cereal-free dog food and in six cereal foods. In none of the analyzed foods *Enterobacteriaceae* were found, including coliforms, *Escherichia coli* and *Salmonella* spp. Bacteria of the genus *Listeria* and *Clostridium* as well as yeasts were also not detected. In conclusion, the evaluated dry dog foods had varied microbiological quality. The detected number of microorganisms may have some implications for long-term consumption of contaminated food. The lack of European Commission standards regarding the permissible amounts of microorganisms in pet food may result in insufficient quality control of these products.

## Introduction

The population of pets is gradually increasing in Europe – an estimated 80 million European households have at least one pet animal (FEDIAF 2020a). It can be said that pets play a particularly important role in the lives of people who regard their pets as "members of the family" (Di Cerbo et al. 2017; Rauktis et al. 2017; McConnell et al. 2019). Due to the growing number of pets in European homes, the pet food market is also developing dynamically. Nowadays, pet food is widespread and used by many animal owners, since it is easy and economical and a freely available way to feed pets throughout their lives. The annual growth rate of the pet food industry is estimated at 2.6% (FEDIAF 2020a).

However, this industry raises controversy and questions about the morality and integrity of production. Safe pet food means that food will not harm animal health or the environment (including people) when it is prepared and consumed in accordance with its intended use (ISO 22,000:^2018^). According to reports in the Rapid Alert System for Food and Feed (RASFF) system (2018), pet food can be a significant source of many hazards associated with biological, physical or chemical agents in animal feed that are reasonably likely to cause illness or injury for pets in the absence of adequate production control.

The dominant type of pet food available on the market is dry food formulated in kibbles, as it is easily stored and effective in satisfying nutritional needs of the animal. According EU regulations (EC 767/2009) when complete pet food is fed over an extended period (i.e. covering the whole period of the life stage) as the only source of nutrients, it will provide all the nutritional needs of the particular animals of the given species and physiological state for which it is intended. Therefore, it is necessary to evaluate the quality of the pet food, and a number of studies have been conducted to test dog food (Hill et al. 2009; Rolinec et al. 2016; Alvarenga et al. 2018; Meineri et al. 2019).

Contemporary pet food formulations use various foods as their main ingredients, including different plant-based ingredients. There is a substantial interest in the topic of grain-free trend in pet food sector (Meineri et al. 2020). The presence or absence of cereals may affect the nutritional value of the finished product (Pezzali and Aldrich 2019; Kazimierska et al. 2020), thus it is worth paying attention to these ingredients when choosing a dog food. However, it seems that “grain-free” is a marketing term rather than scientific definition. On the basis of the Encyclopedia of Grain Science (Wrigley 2004) grains include, among others, green beans, sugar peas, lupins, amaranth, and linseeds. Therefore, when it comes to the presence or absence of cereals in the composition, more scientifically appropriate phrase seem to be “cereal-free”.

Dry dog foods are usually processed at temperatures of 80–160 °C under high pressure (Crane et al. 2010; Meineri et al. 2019). The purpose is to reduce waste, increase the stability of the product and improve the digestibility of carbohydrates. Moreover, high temperatures significantly reduce the number of pathogenic bacteria (Macías-Montes et al. 2020). Nevertheless, Leiva et al. (2019) pointed out that thermal process to improve the safety of pet food is not applicable if the final product is contaminated later in the process. The occurrence of pathogenic microorganisms is associated with cross-contamination and a deviation from good manufacturing practices (GMP) (Meghwal et al. 2017).

Good microbiological quality of food is the main factor, besides the nutritional value of the food, for the production of healthy and safe food (Chlebicz and Śliżewska 2018). Its importance is attributed to the pathogenic microorganisms and non-pathogenic microorganisms which play a role as food hygiene indicators (Hinton 2000). Many research reports have exposed pet food quality problems and their influence on human and animal health. In recent years notifications of pathogenic microorganisms (bacteria, fungi, and the toxins that they produced) constituted about 20% of all notifications for food and feed in RASFF, showing in particular the presence of *Salmonella*, *Listeria*, *Escherichia* and others (Pigłowski 2019; RASFF 2020).

A good example of problems with the microbiological quality of dry dog food is a study conducted in 2006–2008 in the United States (Behravesh et al. 2010), which showed considerable contamination of dry dog foods with *Salmonella,* which may be an under-recognized cause of human infection, especially in young children. *Salmonella* is the most important biological hazard in animal feed; materials and compound feed can be both a vector and a reservoir of *Salmonella* spp. (Maciorowski et al. 2006; Behravesh et al. 2010). The most common source of this pathogen are protein-rich raw materials used to prepare livestock feed (Rönnqvist et al. 2018; Minh et al. 2020). In recent years there have been several other documented *Salmonella* contaminations in pet food and treats (Finley et al. 2006; Adley et al. 2011; Li et al. 2012; Lambertini et al. 2016).

Processed pet food has also been reported to contain other microbial pathogens, such as *Listeria*, *Enterobacteriaceae* and *Campylobacter* (Nemser et al. 2014; Nilsson 2015; Baede et al. 2017; Bree et al. 2018; Hellgren et al. 2019; Nüesch-Inderbinen et al. 2019). The level of contamination of feed by *Clostridium* species is an indicator of soil contamination and hygienic conditions during their production and circulation (Maciorowski et al. 2007). Pathogenic *Clostridium* spp. strains may be an important enteropathogenic agent for animals and their different toxins may cause enteritis and enterotoxaemia (Wojdat et al. 2005).

Another risk factor for animal food safety is the presence of fungi and mycotoxins (Silva et al. 2018). Knowledge on food and feed in relation to fungi is critical in assessing the risk of contamination with mycotoxins (Martins et al. 2003). Some studies have reported that the presence of these substances in pet foods can cause significant harm to pet health, with both acute and chronic types of intoxication depending on the contamination and duration of exposure (Gazzotti et al. 2015). Dogs are particularly sensitive to the acute hepatotoxic effects of aflatoxins (Martins et al. 2003).

The aim of this study was to evaluate dry food for adult dogs, with particular reference to: (1) the nutritional value with respect to nutritional guidelines for dogs, (2) comparing the nutritional value of dog foods with particular emphasis on the division into cereal and cereal-free foods, and (3) evaluating their microbiological safety.

## Materials and methods

### Sampling

In order to evaluate a representative selection of the different types of dry dog food available on the European market, products were selected based on database of all products intended for standard maintenance and for different dog breed sizes (small, medium, large) available on the local market and depending on the presence or absence of cereals in the composition. In total, the research material consisted of 36 commercial dry extruded complete food formulated for adult dogs, including 27 international and 9 local brands, bought locally from a range of commercial suppliers and pet food supermarkets. The size of the packages ranged from 500 g to 2 kg.

Key nutritional information provided on the label was recorded such as macronutrient content (percentage protein, fat, moisture, ash, and fiber, as fed) alongside the country of origin and batch number. The composition of the main components of cereal-free dog foods (no 1–17) is shown in Table 2, and the components of cereal foods (no 18–36) in Table 3. All samples were packaged in sealed bags. Representative samples for chemical analysis were collected from each of the three batches of each product The samples were then ground into a powder using a laboratory mill (KNIFETEC 1095, Foss Tecator, Höganäs, Sweden) and placed in sterile containers marked with successive symbols (no 1–36). To prevent cross contamination, the laboratory mill was cleaned and vacuumed between samples. About 200 g each of the milled samples was used for chemical analysis. Three measurement replication was conducted. The remaining milled samples were stored in the individual sealed containers at 4 °C until required for further analysis (microbial evaluation, within two weeks after purchase). The numbering of the thirty-six dog foods tested is consistent in all tables and figures.

### Nutritional quality

#### Proximate analysis

Dry matter (DM), crude protein (CP), crude fiber (CF), ether extract (EE) and crude ash (CA) were measured to assess the nutritional quality of the tested pet food. All tests were performed using ISO 17,025 (2017) accredited methods based on AOAC (2019). To determine dry matter, samples were dried at 105 °C to a constant weight. Crude protein (N × 6.25) was identified by the Kjeldahl method, using a Büchi Scrubber B414 unit and a Büchi 324 distillation set (Büchi Labortechnik AG, Flawil, Switzerland). Crude fat (as ether extract) was identified by traditional Soxhlet extraction method with diethyl ether. Crude fiber was determined as the residue after sequential treatment with 1.25% H_2_SO_4_ and with 1.25% NaOH using an ANKOM^220^ Fiber Analyser (ANKOM Technology, New York, NY, USA). Crude ash was measured by burning in a muffle furnace at 580 °C for 8 h. Nitrogen-free extract (NFE) were determined by the difference between the original weight of the sample and sum of the weights of its moisture, crude protein, crude fat, crude ash and crude fiber as determined by their appropriate analysis.

1 $$NFE\left(wet\;basis\right)(\%)=100-(\%moisture+\%CP+\%EE+\%CA+\%CF)$$

The results are expressed as g per 100 g DM. Levels of CP and EE were compared with recommended amounts of this nutrients for adult dogs determined by the FEDIAF (2020b).

#### Energy value

On the basis of identified chemical composition, metabolizable energy (ME, kcal/100 g DM) of the foods was calculated, according to the predictive equation by the National Research Council (2006), using 4-steps calculation.

Additionally, nutrient (N) ratio was determined as the overall energy contribution percentage that each macronutrient brought to each diet. The crude protein – crude fat – total carbohydrate (CP:EE:NFE) profile and energy intake ratio from each macronutrient was determined from the calculated energy value of foods using Atwater factors (ME), specifying what percentage of total energy is from particular nutrient:

2 $$N:\;ME\;ratio\;\left(\%\right)=\frac{N\times kcal\;from\;1g\;N}{ME}\times100\%$$

### Microbiological analysis

Dog foods were examined according to standards dealing with microbiology of food and feeding stuffs (ISO 7218 2008). Preparation of samples and dilutions for microbiological tests were made in accordance with standard ISO 6887–1:^2017^–5. The research included: determination of the total aerobic mesophilic bacteria count (TAMBC, ISO 4833 2013), enumeration of coagulase-positive staphylococci (CPS) (*Staphylococcus aureus* and other species) (ISO 6888 1999), detection and enumeration of *Enterobacteriaceae* (ISO 21,528 2017), detection and enumeration of presumptive *Escherichia coli* (ISO 4832 2007), enumeration of beta-glucuronidase-positive *Escherichia coli* (ISO 16,649 2004), detection, enumeration and serotyping of *Salmonella* spp. (ISO 6579 2017), detection and enumeration of *Listeria monocytogenes* and of *Listeria* spp. (ISO 11,290 2017), enumeration of *Clostridium perfringens* (ISO 7937 2005) and enumeration of yeasts and molds (ISO 21,527 2009).

Results of the analysis of the presence and quantity of microorganisms were interpreted in accordance with the standards of microbiological testing of food and feeding stuffs (ISO 7218 2008). The number of all microbial colonies was determined according to the formula (ISO 7218):

3 $$N=\frac{\sum C}{V\times 1.1\times d}$$

where:

*C* – total colonies on two selected plates from two successive dilutions, of which at least one contains a minimum of 10 colonies;

*V* – volume of inoculum applied on each plate, in mL;

*d* – dilution corresponding to the first dilution obtained (*d* = 1 when the undiluted sample is tested).

In turn, the number of colonies of identified microorganisms was determined according to the formula (ISO 7218):

4 $$a= \frac{b}{A}\times C$$

where:

*b* – the number of colonies meeting the identification criterion among the number *A* of identified colonies;

*C* – total number of suspect colonies counted on plate.

During the identification of microorganisms, methods indicated by relevant standards were used as well as macroscopic characteristics on special media, Gram staining and microscopic observation.

### Statistical analysis

One factorial analysis of variance (ANOVA) and principal component analysis (PCA) were carried out using the STATISTICA v13.0 software (TIBCO Software Inc., Palo Alto, CA, USA). The significance of differences between the means was assessed using the Tukey test at p = 0.05.

In order to compare the nutritional value of the dog foods, we determined their composition (CP, EE, CF, CA, NFE, ME). The percentage of a given nutrient or metabolic energy in the profile is expressed by an arithmetic mean converted into units on a 9-point scale. For profile comparison, Cohen’s profile similarity coefficient *r*_*c*_ was used, calculated based on the following formula (Cohen 1969):

5 $$r_{C} = \frac{{\sum\limits_{i = 1}^{n} {A_{i} B_{i} + nm^{2} - m\left( {\sum\limits_{i = 1}^{n} {A_{i} + \sum\limits_{i = 1}^{n} {B_{i} } } } \right)} }}{{\sqrt {\left( {\sum\limits_{i = 1}^{n} {A_{i}^{2} + nm^{2} - 2m\sum\limits_{i = 1}^{n} {A_{i} } } } \right)\left( {\sum\limits_{i = 1}^{n} {B_{i}^{2} + nm^{2} - 2m\sum\limits_{i = 1}^{n} {B_{i} } } } \right)} }}$$

where:

*A*_*i*_, *B*_*i*_ – unitarized values of traits included in the compared profiles A and B;

*n* – number of traits in the profile;

*m* – midpoint of the ranking scale.

This coefficient value was measured in the range -1.00 to 1.00, and its interpretation depends on the value: x ≥  + 0.75 (high similarity); + 0.75 > x >  + 0.30 (moderate similarity); + 0.30 ≥ x ≥ -0.30 (no similarity); -0.30 > x > -0.75 (moderate dissimilarity); x ≤ -0.75 (high dissimilarity). The closer were the values of *r*_*c*_ to boundary values (1/-1), the stronger was the evaluated similarity/dissimilarity. Inter-profile analysis was conducted using MS Office 2017.

## Results

### Nutritional value and adequacy

Significant differences were discovered in the proportion of the evaluated nutrients, depending on the food. Significantly greater amounts of protein, fat, ash and fiber were found in cereal-free products. Of all the foods analyzed, significantly more protein was found in cereal-free foods 5, 9, 15, 16 (from 38.07 to 38.97 g/100 g DM, Table 1). The lowest levels of protein were in the cereal-free foods 13, 14 (21.95 and 22.41 g/100 g DM) and the cereal foods 22, 25, 36 (21.40 to 22.27 g/100 g DM). Based on FEDIAF (2020b) daily requirements, all 36 dry foods for adult dogs presented higher protein concentrations than the recommended minimum levels (18 g/100 g DM) (Table 1), considering an energy intake of 110 kcal/kg BW^0.75^ for dogs with moderate activity (1–3 h/day). The average content of proteins was significantly higher in cereal-free foods than in cereal foods.

Based on FEDIAF (2020b) daily requirements, all 36 dry foods for adult dogs presented higher fat concentrations than the recommended minimum levels (Table 1). Significantly more EE was found in examined cereal-free food no 17 (21.39 g/100 g DM) and the least in cereal foods 23 and 24 (6.31 and 6.76 g/100 g DM). The average content of this nutrient amounted to 15.13 g/100 g DM in cereal-free foods, which is almost three times the recommended minimum levels. In cereal foods the average content of EE amounted to 10.75 g/100 g DM.

Significantly more CA were found in cereal-free foods 4 and 16 (9.92 and 9.88 g/100 g DM) and the least in cereal-free food 11 (4.80 g/100 g DM).

Significantly more CF was found in cereal-free food 6 (15.14 g/100 g DM) and the least in cereal food 36 (1.71 g/100 g DM). In this case, the average content of this nutrient was significantly higher in cereal-free foods and amounted to 8.57 g/100 g DM. Also worth paying attention to, is the ratio of the amount of CA and CP. The dog foods with the lowest amount of ash were also characterized by a relatively low protein content. On the other hand, the tested foods with the highest amount of protein (5, 9, 15, 16) had a large amount of CA.

The main component of DM appeared to be nitrogen-free extracts, consisting of simple sugars, starch, dextrins and organic acids. NFE content in the tested dog foods ranged from 17.74 to 54.28 g per 100 g DM. The difference between the averages in cereal and cereal-free foods varied significantly. The average content in cereal foods amounted to 44.68 g/100 g DM and to 31.50 g/100 g DM in foods labeled as cereal-free.

Cereal-free and cereals food did not differ significantly in the means of metabolizable energy content (369.4 kcal ME/100 g DM and 369.5 kcal ME/100 g DM, respectively). Significantly higher metabolizable energy value was found in cereal food 31 (407.4 kcal ME/100 g DM), and the lowest in cereal food 33 (319.4 kcal ME/100 g DM) (Table 1).

The differences in the levels of individual components were assessed (ANOVA), but additionally a comparative analysis of the nutritional profiles of the tested foods overall (Cohen's profile similarity coefficient) was performed. Clear differences in the similarity of the food profiles was observed depending on the presence/absence of a component of cereal origin in the food, which is also shown by the aforementioned statistical significance of contrast (Tables 2, 3, 4).

The number of comparisons of nutrient profiles for cereal-free foods was 136, and their differences prove a clear variation (Table 2). A lack of similarity (lack of color) was found 40 times, and dissimilarity coefficients (dissimilarity—red color) were found 50 times. This means that the remaining Cohen's profile similarity coefficients (36) were above 0.3 (green) and reflected similarity, i.e. graded conformity (shades of green) of nutrient profiles in nutrient content and energy value.

**Fig. 1:**
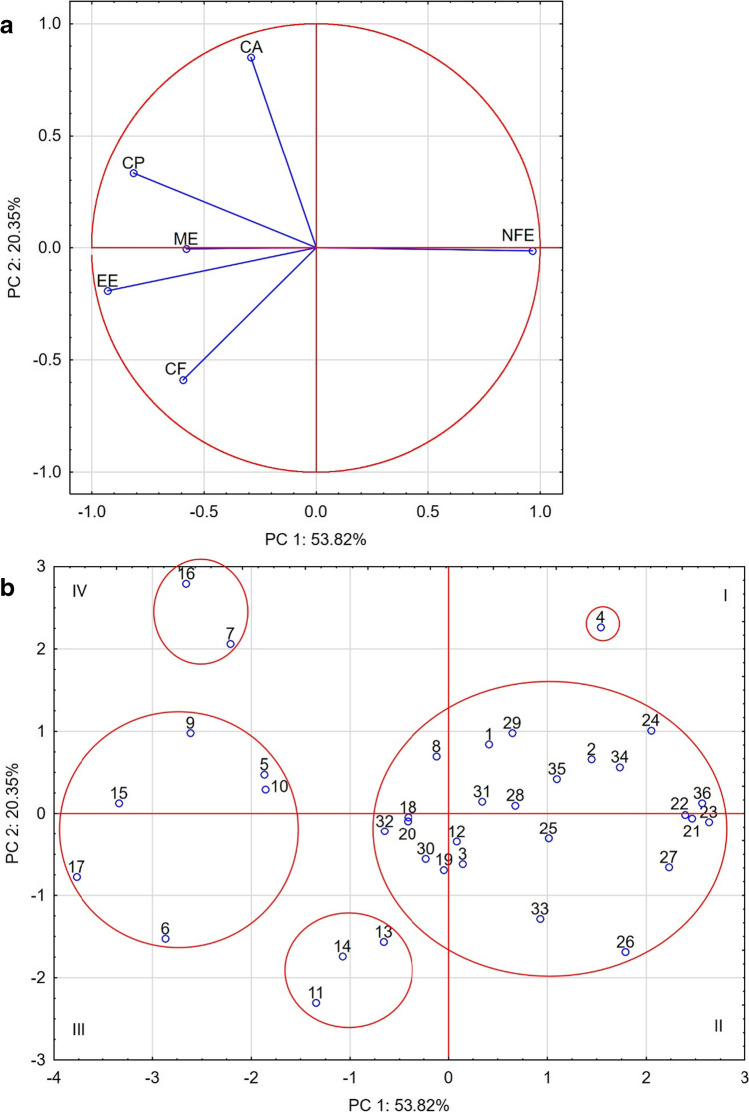
Biplot based on first two principal component axes for nutritional value and metabolic energy of dog foods (**a**) and distribution of 36 commercial dog foods based on the first two components obtained from principal component analysis (**b**)

The foods most often showing dissimilarity were 1, 2 and 4, while showing a high mutual similarity coefficient (r_c_ > 0.75). These foods were the only ones to contain sweet potatoes, peas and potatoes among the main ingredients. Their similarity was also confirmed by PCA analysis, placing these foods in the first quadrant (Fig. 1b). Foods 1 and 2 were also part of the largest group of foods with increased NFE content and reduced content of other components, especially CP and EE (Fig. 1a). This group is dominated by foods containing cereals, and at the same time foods with this component did not appear in any of the other defined groups.

In group of cereal-free foods, numbers 15 and 17 showed most frequent similarity of their profiles to other foods in this group. They were located in the 3rd and 4th quadrant, with reduced NFE but increased CP, EE and CF (Fig. 1a, b). The PCA analysis allowed to distinguish two more different groups within the cereal-free foods, the first one for foods 7 and 16 and the second one for 11, 13 and 14. The foods in both groups had less NFE, but the former was richer in CA and the latter in CF (Fig. 1a, b). This analysis also confirms a clear negative correlation between NFE level in food and ME.

A much more homogenous group of foods were those with cereal components (Table 3). The aforementioned table shows a clear predominance of green, confirming the most frequent occurrence of moderate similarity or high similarity of nutritional profiles in this group of foods, as confirmed by the PCA analysis which puts them in one group (Fig. 1b). The only foods in this group that show profile dissimilarity in relation to the other foods in this group are foods 32 and 33, although they do show a moderate mutual similarity (r_c_ = 0.66) (Table 3). The common element in their composition, apart from rice, chicken fat and salmon fat, is the presence of apples. The PCA analysis places these feeds in the largest group of feeds, in the 3rd and 2nd quadrant, respectively.

The observed relationships regarding the similarity of the nutritional profiles of the groups with and without cereal products are also visible in the similarity of the profiles in both these groups (Table 4). Dog foods 1, 2, 3 and 4, without a cereal component, containing sweet potatoes, peas, potatoes, flaxseed and beet pulp showed high similarities with cereal foods which also contained flaxseed (foods 23 to 36) and beet pulp (foods 21 and 22). This is demonstrated by the Cohen's similarity coefficients in the individual designated ranges. Thus, for these four cereal-free foods, no coefficient of high dissimilarity was observed and the moderate similarity (light red) occurred 3 times.

17 dissimilarity coefficients (lack of color) were observed, which means that the remaining 56 similarity coefficients of food profiles denoted moderate or high similarity. A less frequent similarity with cereal foods and – when the similarity occurred – lesser intensity (moderate) was observed for foods 12, 13 and 14. Two of them, 13 and 14, contained larvae of *Hermetia illucens* as one of the basic components. Despite the fact that these foods (1, 2, 3, 4, 12, 13, 14) showed varying similarity to cereal feeds, they also showed moderate dissimilarity within their entire group. The PCA analysis of foods 1, 2, 3 and 12 includes them in cereal foods, while the rest are classified as two separate groups.

The proportions of energy substrates in the dog's diet were calculated and compared to those suggested by Hewson-Hughes et al. (2013). The proportion of energy derived from protein ranged from 23 to 32% in cereal foods and from 22 to 41% in cereal-free foods (Table 5). Fats in the tested foods constituted of 21–48% in cereal-free foods and 16–35% in cereal foods, and carbohydrates of 18–49% in cereal-free foods and 37–57% in cereal foods of the energy value of the tested foods.

### Microbiological safety

The TAMBC in the analyzed dry dog food ranged from 2.7 × 10^2^ to above 3 × 10^7^ cfu/g (Table 6). In eighteen (50%) tested foods, contamination above 10^4^ cfu/g was recorded, of which five (14%) foods (2, 8, 13, 19, 26) had contamination above 10^6^ cfu/g.

In five (14%) of the tested dog foods (12, 13, 14, 17, 18), the presence of staphylococci was found; however, CPS was not found (Table 6). In the test for the presence of CPS using Giolitti-Cantoni Broth (Oxoid) a positive reaction was noted by the blackening of the medium. Samples were then streaked onto Baird-Parker Agar medium with RPF supplement (Oxoid); however, none of the CPS was detected. In the test for enumeration of staphylococci using a pour plate method and Baird-Parker Agar medium with RPF supplement (Oxoid), the presence of staphylococci was detected in the same five (14%) dog foods. The number of isolated staphylococci ranged from 3.6 × 10^1^ to 8.3 × 10^2^ cfu/g. None of the isolated staphylococci was coagulase positive.

Mold presence was recorded in one cereal-free dog food (no 9) and in six cereal foods (18, 19, 21, 23, 33, 36), which in total is 19% of the tested foods. The level of contamination ranged from 1 × 10^2^ to 1 × 10^3^ in cereal-free dog food, while in cereal foods from 1 × 10^3^ to 1 × 10^5^ cfu/g. The identified fungi belonged to the genus *Aspergillus* and *Rhizopus*.

In none of the analyzed foods was *Enterobacteriaceae* family found, including the coliforms, *Escherichia coli* and *Salmonella* spp., bacteria of the genus *Listeria* and *Clostridium* as well as yeasts were also not detected (ND) (Table 6).

## Discussion

Nutrition of pets is central for their health and well-being. FEDIAF developed the guideline to good practice for the manufacture of safe pet foods based on EU regulations (EC 2073/2005) that European pet food manufacturers should follow. The scope of the guide includes the production, storage and distribution of dry, semi-moist and wet pet food and dog chews, as well as imports into the EU (FEDIAF 2018).

### Nutritional quality

The FEDIAF guidelines are the only recommendations used in Europe for the chemical composition of pet foods and their recommended minimum level and nutritional maximum limit. The levels given in the FEDIAF guide reflect the amounts of essential nutrients in commercial pet foods that are required to ensure sufficient and safe nutrition in healthy dogs when consumed over time. The recommended minimum levels of macronutrients for adult dogs concern only protein and fat, with levels set at 18 g and 5.5 g per 100 g DM respectively, considering a daily energy intake of 110 kcal/kg BW^0.75^. In our study, all tested foods met the recommended minimum levels for total protein and fat.

Those nutrient levels are minimum recommended allowances for commercial pet food, not minimum requirements or optimal intake levels (FEDIAF 2020b). For example, Case et al. (2011) claim that nutrient content should be not less than 26 g of CP, not less than 15 g of EE and not more than 5 g of CF per 100 g DM. In that case, the tested foods compared much worse: 13 of the foods (36%) had less than 26 g/100 g DM of CP, 25 foods (69%) had less than 15 g/100 g DM of EE and only 10 foods (28%) had less than 5 g/100 g DM of CF. There is a controversy on the optimal level of several nutrients required by adult dogs, especially on the level of protein. However, it is one of the most important nutrients in a dog diet. There are studies suggesting that long-term feeding of dogs with high protein food content is associated with negative microbial and metabolic profiles (Gebreselassie and Jewell 2019). Dietary fiber may impact on kibble texture and is an important component in the production of extruded pet foods (Monti et al. 2016). Moreover, fiber is used to reduce energy value and have an impact on gut health (Kawauchi et al. 2011; Fischer et al. 2012). On the other hand, too large amounts of fiber may reduce the digestibility of food. Commercial pet foods are available in a variety formulations because once recommended levels for macronutrients are guaranteed, the manufacturers may include them in convenient amounts. It is important that along with the amounts of protein and fat, a product must provide enough essential amino acids and fatty acids.

The content of nutrients is one issue. Another is CP:EE:NFE profile and energy intake ratio from each nutrient. Nutrition standards for pets do not provide recommendations for the amount of energy from each nutrient. Hewson-Hughes et al. (2013) proposed the macronutrient CP:EE:NFE ratio for the domestic dog diet at approximately 30:63:7, based on intakes of dogs with prior experience of the respective experimental food combinations. Interestingly, in later research (Bosch et al. 2015) nutrient intake has been compared between domestic dogs and wolves. It was found that the wolf diet consists of more protein and is characterized by less total carbohydrate intake (CP:EE:NFE = 54:45:1% by energy). Furthermore, the authors asserted that the dogs preference for lipid-rich diets is a trait that does not stem from the early domestication of the dog but rather has evolved during the evolution of its forebear, the wolf (Bosch et al. 2015).

Our results in terms of fat and carbohydrates deviate significantly from assumptions made by Hewson-Hughes et al. (2013). In each of the tested foods, the percentage of energy from fat did not exceed 48% (average 30%). In turn, the percentage of energy from carbohydrates in each food is more than 21% (average 41%), which confirms the widely held opinion that dry extruded foods contain large amounts of carbohydrates. However, excluding all carbohydrate sources from dog's diet, which contain plant products rich in minerals and vitamins, can lead to the need for supplementation. In addition, the production process of extruded dry food requires materials containing starch and dietary fiber that play a structural role and regulate the course of physical changes during the extrusion process. Starch contributes to both expansion and binding in the final product. Cereal (e.g. rice, barley, oats and sorghum) and cereal by-products (e.g. flour, bran) are largely used for pet food. Normally these by-products contain a good quantity of fiber (Rokey et al. 2010).

Only the energy derived from protein agrees with the assumptions of Hewson-Hughes et al. (2013) study (average 29%); the protein-fat-carbohydrates profile in analyzed dry dog food is 29:30:41. It is worth noting that cereal-free foods had a better CP:EE:NFE profile (32:35:33) than cereal foods (27:25:48), in comparison to the optimal ratio of 30:63:7 proposed by Hewson-Hughes et al. (2013). The results show that manufacturers replace fats, valuable for dogs, with vegetable carbohydrates. Even if dogs are well adapted to digest a high-starch diet (Axelsson et al. 2013), it does not mean that the amount of carbohydrates in the analyzed dry foods should constitute the largest part of the food.

### Comparing the nutritional values in cereal and cereal-free foods

This study showed a considerable differentiation in the similarity of the nutritional profiles of foods depending on the presence or absence of a cereal component in the dog food. Statistical differentiation of the means of contrasts indicates significant differences between the content of individual nutrients (except for DM content) in the tested cereal-free and cereal dog foods.

Some feed ingredients used commonly in pet food formulations have been described elsewhere (Donadelli et al. 2019; Corsato Alvarenga et al. 2020). Traditional commercial dry pet foods often contain cereals due to their dependable supply and low price (Yamka et al. 2005). However, more recent dog foods often contain cereal-free ingredients (such as legumes, potatoes, sweet potatoes and tapioca) which can be a more beneficial source of carbohydrates fraction for dogs, with potential beneficial effects in improving the health status of pets (e.g. prebiotic effect, antioxidant activity, reducing the risk of colon cancer; De Godoy et al. 2013).

The tested cereal-free foods (1–17) contained as the main source of plant carbohydrates: potato (6), sweet potato (6) and pea (5). In turn, in the tested dog foods with cereals (18–36) the main sources of carbohydrates were: rice (10), barley (3), brown rice (2), oats (2) and sorghum (2). Potatoes, sweet potatoes and legumes, usually found as carbohydrate sources in cereal-free foods, are a good protein and starch source, and if processed properly can be an effective ingredient in monogastric animals diets (Corsato Alvarenga et al. 2020). Compared to most other vegetables, legumes are relatively high in protein. Whole peas are considered a high-quality addition to dog food. They provide carbohydrates, dietary fiber, and small amounts of beneficial vitamins. In dog nutrition, the addition of peas to the food may affect the fecal microbiome in healthy dogs (Sandri et al. 2019). Peas also contain a noticeable quantity of protein. In our study, foods without cereal component, which usually include legumes in their main ingredients, had a higher average content of CP and CF than cereal foods. Cereal-free foods also had a higher average content of EE than cereal foods, which may be due to, among others, the main plant ingredients used. Most of the tested cereal foods contained rice as the main plant component and most cereal-free foods contained peas, which are marked by higher amounts of fat than rice (Tulbek et al. 2017; Sandri et al. 2019; Ismagilov et al. 2020).

Pet foods often rely on the use of by-products, such as potato fiber, a by-product of potato starch manufacture, and a functional dietary fiber source in dog foods (Panasevich et al. 2013). In our study, the cereal-free foods containing potatoes or potato by-products had significantly higher CF-content, which is in line with the aforementioned study. Furthermore, a potato-based diet affects the fecal microbiome in dogs, improving the molar proportion of lactic acid and decreasing pH and N-NH_3_ concentrations (Sandri et al. 2020). In turn, sweet potato, which contains from 1 to 9% of protein, is a perfect source of natural health-promoting compounds, such as β-carotene and anthocyanins (Bovell-Benjamin 2007; Mohanraj 2018) which makes it a frequent ingredient in dog food.

Cereals supply most of all energy and carbohydrates, and make up 25–60% of DM in cereal dry dog foods. Dogs are able to digest starch, but the composition and structure of starches from grains may affect their digestibility. Fortes et al. (2010) concluded that the maize, broken rice, sorghum and millet had better digestibility and greater metabolizable energy for dogs than brans (wheat and rice). Twomey et al. (2003) reported that the digestibility of fat, protein and energy in the sorghum-based diets was lower compared to the rice-based diet. Simultaneously, the quality of feces seen in dogs fed with sorghum improved, indicating that sorghum can be a replacement of rice as the primary cereal in dry dog foods. Rodehutscord et al. (2016) studied the chemical constituents of different genotypes of cereal grains. Analyzed cereals (barley, maize, oats, rye, triticale and wheat) consisted of 68.9–82.9% of NFE, which may explain the statistical significance of contrast for NFE in our study. Interestingly, a study conducted by Pezzali and Aldrich (2019) has shown that dogs prefer cereal-free over foods with ancient grains in the palatability assessment of dry food.

A study evaluating digestibility and fecal traits in cereal and cereal-free foods for dogs (Chiofalo et al. 2019) concluded that the cereal-free diet had higher apparent nutrient digestibility of protein and fat and more stable large intestinal fermentation of carbohydrates compared to the diet with cereals. This enabled dogs to more efficiently use nutrients from the diet, thus requiring less food. Comparing this data with the results of our analyses, where cereal-free foods obtained a higher average CP (+ 21%), EE (+ 40%) and CA (+ 11%) content, and a lower amount of NFE (- 30%), it could be considered that a cereal-free diet for dogs may be more beneficial. Further research in this area seems to be required.

### Microbiological safety

Currently, there are no strict regulations on maximum limits of particular bacterial and fungal contamination for pet foods. According to EU law, “the feed business is primarily responsible for feed safety” (EC 183/2005). The microbiological requirements currently in force do not allow the presence of *Salmonella* in 5 samples of feed weighing 25 g, and limit the number of *Enterobacteriaceae* in feed materials of animal origin from 10 to 300 cfu/g in 2 out of 5 samples of a tested batch (EU 142/2011). Moreover, EC Regulation (2073/2005) on the general rules of feed hygiene, does not apply to retail pet food. According to FEDIAF (2018), the list of biological hazards in dry pet food that are reasonably likely to cause illness or damage to animals in the absence of monitoring include *Enterobacteriaceae*, pathogenic *Escherichia coli*, *Salmonella*, *Staphylococcus aureus*, *Listeria monocytogenes*, *Clostridium perfringens*, *Clostridium botulinum*, *Aeromonas*, *Campylobacter*, molds and yeasts.

Food contamination by microorganisms is a risk factor to animal health (Kukier et al. 2012; Tessari et al. 2014; Bilung et al. 2018; Leiva et al. 2019). Raw food materials used in feeds of both animal and plant origin may be the initial stage of microbial contamination of food products (Ruzauskas et al. 2005), during their storage, transport, production of food, packaging and / or storage of the final product (Girio et al. 2012). Assessment of the microbiological status of foods is therefore an important element of the nutritional safety of animals. Moreover, there are documented instances of illnesses in dog caregivers resulting from contact with a contaminated product (Behravesh et al. 2010; Imanishi et al. 2014). It should be emphasized that food can be an unrecognized source of infection, especially in young children and the elderly (Behravesh et al. 2010; Stull et al. 2013; Imanishi et al. 2014).

The probability of the occurrence of pathogenic microorganisms and their toxic metabolites in food may increase with the total number of microorganisms present in a given product. Although there are currently no regulations that specifically limit the values of TAMBC in dog food, according to research by Kukier (2012) conducted on the microbiological quality of animal feed, TAMBC in feed should not exceed 10^6^ cfu/g. In this study, in the 36 dry dog foods tested, the total number of microorganisms ranged from 2.7 × 10^2^ to above 3 × 10^7^ cfu/g. A total count of microorganisms above acceptable 10^6^ cfu/g according to Kukier (2012) was recorded in five (14%) of the dog foods. In contrast, a study by Hołda et al. (2017) noted much lower microbial contamination. Among 20 dry dog foods tested, typical growth of aerobic bacteria was detected in 15 (75%) products ranging between 1.0 × 10^1^ to 2.7 × 10^2^ cfu/g. Leiva et al. (2019) found a mean of 382 cfu/g of total mesophilic bacterial count in puppy foods collected from 2012–2018. The authors reported that the acceptable limit for TAMBC is 5 × 10^4^ cfu/g. However, the obtained results of microbiological tests for the presence of TAMBC in our research cannot be compared to the legal provisions regulating the degree of microbial contamination, because currently there are no legal provisions specifying the permissible level of total mesophilic bacteria.

Microorganisms present in cereals (bacteria belonging to the families: *Pseudomonadaceae*, *Micrococcaceae*, *Lactobacillaceae*, *Bacillaceae*, and fungi, like: *Alternaria* sp., *Aspergillus* sp., *Cladosporium* sp., *Penicillium* sp., *Fusarium* sp., *Rhizopus* sp.) can affect the safety, quality and functional properties of the grains and thus the quality of the dog food made from these cereals (Los et al. 2018; Witaszak et al. 2020). Thus, it can be assumed that the presence or absence of cereals in the food may affect the microbiological quality of the product. In our study, out of five dry dog foods where the total count of microorganisms was above the acceptable degree, three were cereal-free foods (60%) and two were cereal foods (40%). Therefore, it can be assumed that the carbohydrate source did not affect the number of TAMBC.

There are no standards specifying the permitted number of staphylococci in animal feed. However, taking into account the pathogenicity of these bacteria, enumeration of staphylococci, especially *Staphylococcus aureus* and other CPS, was analyzed in the presented work. In five (14%) tested dry dog foods the presence of staphylococci was detected, of which four out of five (80%) were cereal-free foods. Our sample size is too small to draw far-reaching conclusions comparing cereal-free and cereal foods. Nevertheless, some attention is needed on this issue and examination should continue in subsequent studies on a larger scale. The total number of staphylococci ranged from 3.6 × 10^1^ to 8.3 × 10^2^ cfu/g and none of the isolated staphylococci was CPS. Other researchers have also analyzed animal feeds for the presence of staphylococci. For instance, Galvao et al. (2015) found staphylococci in less than 2% of 108 tested foods. However, a similar to our study bacterial count was recorded, ranging from 3.5 × 10^1^ to 4.5 × 10^1^ cfu/g. Again, as in our research, none of the staphylococci was coagulase positive. In another study, Weese (2005) found the presence of *S. aureus* in one of the 25 tested raw foods (4%) at over 10^5^ cfu/g. In turn, Freeman et al. (2013) examined 26 bully sticks and found one (4%) methicillin-resistant *Staphylococcus aureus* (MRSA).

The presence of molds in feeds reduces the nutritional value of product and implies a potential risk for animal health, such as mycotoxicosis and immunosuppression (Martins et al. 2003; Ankande et al. 2006). It was assumed that the number of yeasts and molds exceeding 10^4^ cfu/g indicates poor microbiological quality of food and levels exceeding the recommended limits to ensure hygienic quality (GMP 2005). Wojdat et al. (2005) noticed an unacceptable number of fungi in 9% (7 out of 77) of analyzed animal compound feeds. In our study, in 7 out of 36 (19%) tested dry dog foods the presence of mold was reported, ranging from 1 × 10^2^ to 1 × 10^5^ cfu/g. The identified molds belonged to the genus *Aspergillus* and *Rhizopus*. Yeasts were not present in any foods. In contrast, Hołda et al. (2017) recorded a lower level of mold contamination of dry dog food, below 2.0 × 10^1^ cfu/g, and isolated *Aspergillus*, *Penicillium* and / or *Rhizopus* species in five out of 20 tested foods (25%). In a similar study, Bueno et al. (2001) detected fungi in all (100%) 12 samples in commercial dry dog food. The most prevalent genera from a total of 39 isolated strains were *Aspergillus* (67%), *Rhizopus* (42%) and *Mucor* (42%). Also Martins et al. (2003) detected *Aspergillus* (58.3%), *Penicillium* (38.3%) and *Mucor* (38.3%) in dry pet food for dogs, cats and birds, but samples showed low levels of contamination (10^1^ to 10^2^ cfu/g). However, it was dog food that had higher percentage of contamination than the other tested foods. In one of the most recent studies, Witaszak et al. (2020) isolated mycotoxigenic fungi belonging to five genus: *Alternaria* (7%), *Aspergillus* (12%), *Cladosporium* (10%), *Penicillium* (38%) and *Fusarium* (33%), from 38 cat and dog dry foods available on the Polish market. The traditional use of large amounts of plant ingredients (including cereals) by pet food producers, especially in dry product formulas, has greatly increased the risk of mycotoxin poisoning in pets (Boermans and Leung 2007), since the mycotoxins produced by some molds could potentially pose a health risk, in particular where the various stages of the pet food production process were not able to deactivate these fungal metabolites (Bullerman and Bianchini 2007). However, food, feed or other products contaminated with mold fungi do not always contain mycotoxins. Different factors affect the formation of mycotoxins, e.g.: weather conditions, susceptibility of the crop, geographic and seasonal factors, temperature, humidity, cultivation, harvesting, storage and transportation practices, as well as presence or absence of specific nutrients and inhibitors (D'Mello and Macdonald 1997). The results of our research have shown that the presence of yeast and molds was more common in cereal foods (6 out of 7 positive results apply to cereal foods, 86%). Therefore, it can be suggested that cereal foods due to the presence of cereals are a more frequent source of molds and yeast in extruded food for dogs, and thus pose a greater risk to animal health in this regard. However, this requires further research.

It is considered that the number of *Enterobacteriaceae* in animal feeds may not exceed 3 × 10^2^ cfu/g in the tested samples (EU 142/2011). In our research, *Enterobacteriaceae* were not found in any of the dog food samples, unlike Hołda et al. (2017) who identified *Enterobacteriaceae* in twelve out of twenty (60%) dry dog foods with a range from 1.0 × 10^1^ to 2.3 × 10^2^ cfu/g. However, it needs to be remembered that raw diets pose a greater bacteriological threat than dry foods due to the simplistic preparation and no heat treatment.

Although there is no limit set for the presence of *Escherichia coli* in animal feeds, it is considered that their number should not exceed 3 × 10^2^ cfu/g. In this study no contamination of *Escherichia coli* was found in any of the examined dog foods. However, Hołda et al. (2017) isolated 10–50 cfu/g of *E. coli* in four out of twenty dog foods (20%).

Salmonellosis is the second most commonly reported zoonosis in humans in the EU (EFSA 2018; Jansen et al. 2019). The genus *Salmonella* is considered to be the greatest microbiological risk in pet foods (Behravesh et al. 2010). Behravesh et al. (2010) also documenting the first case of human salmonellosis from handling dry dog food. As stated above, according to EU requirements, *Salmonella* should not be present in 25 g of the product tested (EU 142/2011). It can be found both in dry and wet pet food, and presents a unique hazard and a significant challenge to human and pet food producers due to its ability survive in high fat, low moisture matrices. In our research *Salmonella* sp. was not detected in 25 g of dog food. The same result was recorded by Hołda et al. (2017). In turn, Wojdat et al. (2004) found *Salmonella* spp. in 22 of the 2271 (1%) dry foods tested, while Wojdat et al. (2005) isolated *Salmonella* spp. from 10 out of 169 (5.9%) feed tested (6.7% from compound feeds and 4.6% from raw materials). This difference in the frequency of isolation was probably due to the type of food – dry and processed vs compound and raw. According to Lambertin et al. (2016) and Oni et al. (2016) *Salmonella* control during the production of dry pet food is a complex endeavor requiring control of the ingredient quality, one or more microbial reduction steps, controls to avoid potential cross-contamination, and control of moisture. Research conducted by Li et al. (2012), based on checking the level of contamination of raw materials for the production of animal feed, concluded that the microbiological quality has significantly improved, from the first examined period (2002–2006) where *Salmonella* was determined in 12.4% of the tested animal feeds, to the next time period (2007–2009) where *Salmonella* was determined in only 6.1% of the samples. The authors believe that the reason for the improvement in the microbiological state of the feed is the higher microbiological quality of raw materials used for production. The Centers for Disease Control and Prevention (CDC 2012) investigated cases in which human and/or animal illness was associated with exposure to Salmonella-infected pet foods in a total of 49 individuals (47 individuals in 20 states and two individuals in Canada) infected with the outbreak strain of *Salmonella infantis*. Epidemiological and laboratory investigations conducted by officials in local, state and federal public health, agriculture, and regulatory agencies linked this outbreak to dry dog food.

Low-moisture foods are generally considered “lower risk” by food safety program and risk managers supporting food manufacturers and product distributors, as intrinsic factors including water activity limit bacterial growth of food-borne pathogens. Microbiological criteria for ready-to-eat foods (RTE) may have levels below 100 cfu/g in food based on a scientifically valid sampling scheme (CA 2007). European Union adapted these microbial criteria for the verification and control of *Listeria monocytogenes* in RTE foods (EU 2073/2005). EU regulations do not specify requirements for the detection of genus *Listeria* in animal feeds. It is assumed, however, that feeds should meet the requirements specified for food intended for humans. This means that *Listeria* cannot be present in 25 g of the product, or its number must be less than 100 cfu/g (EU 2073/2005), if the manufacturer guarantees that the product will not exceed this level during the entire shelf life. In our study *Listeria* sp. was not detected. All the detected colonies that were isolated were found to belong to the genus *Bacillus*. Similarly, Bilung et al. (2018) examined 32 foods of different types for cats and dogs, and none of the samples were contaminated by *Listeria monocytogenes*. Hołda et al. (2017) had similar observations and also did not find *Listeria* sp. in 20 dog foods tested, whereas Nemser et al. (2014) examined 480 dry foods for dogs and cats, and found *L. monocytogenes* only in one cat food.

Wet foods are more problematic in terms of the presence of *Listeria* or *Salmonella* compared to dry foods. Nevertheless, dog owners should be careful when handling any types dog food products, having regard to the potential risk to human and animal health.

*Clostridium* spp. has been isolated from feeds intended for dogs, cats and horses (Borriello et al. 1983; Broda et al. 1996; Pirs et al. 2008; Gould and Limbago 2010). In this study, microbial contamination by *Clostridium* spp. was not found. However, Freeman et al. (2013) isolated *C. difficile* in one (4%) of 26 bully sticks. Manufacturers should take steps to reduce the possibility of contamination of products with pathogenic bacteria (Nemser et al. 2014).

## Conclusion

All the evaluated dry dog foods met the minimum FEDIAF requirement for total protein and fat content. Cereal-free foods contained significantly more CP, EE, CF and CA than cereal foods. In turn, cereal foods contained significantly more NFE. The dog foods also differed in the ratio of energy from individual nutrients. The cereal-free foods had more optimal PC:EE:NFE profile (32:35:33) than cereal foods (27:25:48), against the optimal ratio of 30:63:7 proposed by Hewson-Hughes et al. (2013). Comparative analysis of the nutritional profile of foods based on the proportions of nutrients and metabolic energy also showed differences between foods depending on the composition. Cereal foods were more similar to each other, probably because the cereals are systematically and botanically more similar than the plant components used in the cereal-free foods.

The evaluated dog foods had varied microbiological quality. Although currently there are no regulations that specifically limit the values of TAMBC in dog foods, too large a quantity of microorganisms in the pet food decreases its quality. Eighteen (50%) of the tested foods had contamination above 10^4^ cfu/g, of which five (14%) had contamination above 10^6^ cfu/g. Moreover, staphylococci were detected in 14% of the tested foods. Despite no CPS, the presence of staphylococci could be an indicator of poor feed hygiene. Furthermore, in seven of the 36 dog foods (19%) mold was found, where the occurrence was more common in cereal foods (6 of 7 positive results were in cereal foods, 86%). Then, it can be suggested that cereal foods, due to the presence of cereals, are a more frequent source of molds and yeast in dried food for dogs, and thus pose a greater risk to animal health in this regard.

In this study we observed different levels of nutritional and microbiological quality of the tested products. The lack of EC standards regarding the permissible amounts of microorganisms in pet food may result in insufficient quality control of these products, which carries health risks for animals as well as humans. Therefore, the results indicate the need for monitoring the microbiological quality of pet food. Future research should be extended to include other types of pet food.

**Table 1:** Chemical composition (g/100 g DM) and energy value (kcal/100 g DM) of the analyzed commercial cereal-free (no 1-17) and cereal (no 18-36) dog food^1^

Item	DM g/100 g	CP	EE	CF	CA	NFE	ME
1	92.85^fghi^	28.14^ijkl^	11.61^cdefg^	5.59^cdefg^	7.78^p^	39.74^efg^	374.1^ijklmn^
2	92.86^ghi^	24.40^ cd^	9.82^bcdef^	5.71^defgh^	7.74^p^	45.21^hijk^	364.4^fghijk^
3	92.45^cde^	29.42^ lm^	12.26^efgh^	7.30^ghijk^	5.92^ cd^	37.56^def^	371.4^hijklmn^
4	92.46^cde^	25.71^def^	8.02^ab^	5.83^defghi^	9.92^t^	43.00^gh^	348.6^bc^
5	94.09^ lm^	38.20^o^	14.26^ghijkl^	7.79^ghijkl^	7.04^ lm^	26.82^b^	374.9^ijklmn^
6	92.85^fghi^	34.23^n^	18.21^o^	15.14^p^	6.37^fgh^	18.92^a^	336.6^ab^
7	93.15^ij^	34.74^n^	17.25^mno^	4.24^bcdef^	9.10^ s^	27.83^b^	406.4^pq^
8	92.37^ cd^	30.25^ m^	11.65^defg^	8.52^jkl^	8.05^q^	33.91^ cd^	353.5^bcd^
9	91.11^a^	38.97^o^	16.47^klmno^	7.59^ghijkl^	7,82^p^	20.27^a^	383.3^mno^
10	91.64^b^	35.41^n^	15.77^jklmno^	7.75^ghijkl^	7.10^mno^	25.62^b^	380.7^klmno^
11	92.53^def^	29.20^jklm^	16.81^lmno^	11.90^no^	4.80^a^	29.83^bc^	359.9^defghi^
12	93.60^ k^	26.05^efg^	14.72^hijklm^	4.12^abcde^	6.07^cde^	42.65^gh^	403.5^pq^
13	96.91^q^	21.95^a^	16.98^lmno^	11.55^mno^	6.49^hi^	39.95^efg^	357.0^cdefg^
14	97.55^r^	22.41^a^	17.62^no^	13.38^op^	6.66^ijk^	37.49^def^	345.7^bc^
15	95.87^o^	38.12^o^	17.98^o^	11.76^no^	7.71^p^	20.32^a^	358.1^cdefgh^
16	97.39^r^	38.07^o^	16.49^klmno^	4.32^bcdef^	9.88^t^	28.64^b^	400.6^pq^
17	93.77^kl^	34.14^n^	21.39^p^	13.23^op^	7.28^no^	17.74^a^	360.7^efghij^
18	92.45^cde^	28.27^ijkl^	14.74^hijklm^	5.94^defghi^	6.74^jk^	36.77^de^	388.9^mnop^
19	96.82^q^	29.27^klm^	12.62^ghi^	7.30^ghijk^	5.87^c^	41.78^fgh^	373.1^ijklmn^
20	92.15^c^	26.26^efg^	15.07^ijklmn^	7.84^ghijkl^	7.30^o^	35.68^de^	374.2^ijklmn^
21	92.45^cde^	22.61^ab^	8.50^ab^	3.05^ab^	6.11^de^	52.19^ mn^	381.3^klmno^
22	93.01^hi^	22.27^a^	8.79^ab^	3.10^ab^	6.24^efg^	52.62^ mn^	381.8^klmno^
23	92.56^defg^	23.90^bc^	6.31^a^	6.35^efghij^	6.54^hij^	49.46^klm^	348.7^bc^
24	94.14^ m^	27.92^ijk^	6.76^a^	3.22^abc^	7.17^mno^	49.07^klm^	370.2^hijklmn^
25	92.82^fghi^	21.40^a^	12.33^fgh^	8.20^ijkl^	7.31^o^	43.59^ghi^	358.9^cdefgh^
26	94.51^n^	22.73^ab^	9.82^bcdef^	8.07^hijkl^	4.93^a^	48.96^jklm^	357.4^cdefgh^
27	91.54^b^	22.58^ab^	9.55^bcde^	3.59^abcd^	5.42^b^	50.41^lmn^	384.9^mno^
28	96.34^p^	25.14^cde^	11.81^efg^	6.59^fghij^	7.19^mno^	45.61^hijk^	369.1^fghijklm^
29	95.95^o^	24.29^ cd^	11.84^efg^	6.52^efghij^	8.56^r^	44.75^hij^	364.9^fghijk^
30	93.57^ k^	27.75^hij^	13.51^ghij^	9.13^klm^	6.68^ijk^	36.50^de^	360.8^efghij^
31	92.68^defgh^	26.39^efgh^	13.90^ghijk^	2.90^ab^	6.43^gh^	43.07^gh^	407.4^q^
32	94.73^n^	29.40^ lm^	13.68^ghij^	9.82^lmn^	7.21^mno^	34.62^d^	355.2^bcde^
33	94.63^n^	27.28^ghi^	8.99^abcd^	12.70^op^	6.14^e^	39.54^efg^	319.4^a^
34	93.78^kl^	26.98^fghi^	8.24^ab^	3.88^abcd^	6.84^kl^	47.86^ijkl^	373.5^ijklmn^
35	93.48^jk^	28.91^jklm^	8.89^abc^	6.39^efghij^	7.08^ mn^	42.23^gh^	359.2^defghi^
36	92.73^efgh^	21.69^a^	8.87^ab^	1.71^a^	6.19^ef^	54.28^n^	391.4^nop^
Contrast							
Cereal-free foods	93.73^a^	31.14^b^	15.13^b^	8.57^b^	7.39^b^	31.50^a^	369.4^a^
Cereal foods	93.70^a^	25.53^a^	10.75^a^	6.12^a^	6.63^a^	44.68^b^	369.5^a^
Recommended minimum level							
FEDIAF (2020b)		18.00	5.50				

*DM *dry matter, *CP* crude protein, *EE* ether extract, *CF* crude fiber, *CA *crude ash, *NFE* nitrogen free extract, ME, metabolizable energy

^a^Means with at least one same letter in the superscripts (a, b, c, …) not differ statistically at P = 0.05 (for all columns separately)

**Table 2:**
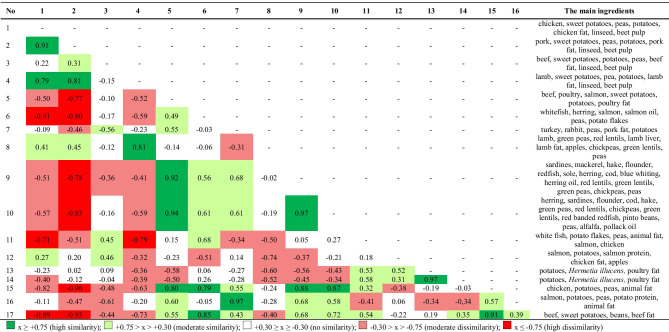
Comparative analysis of the nutritional profile (Cohen’s profile similarity coefficient) for cereal-free foods

**Table 3:**
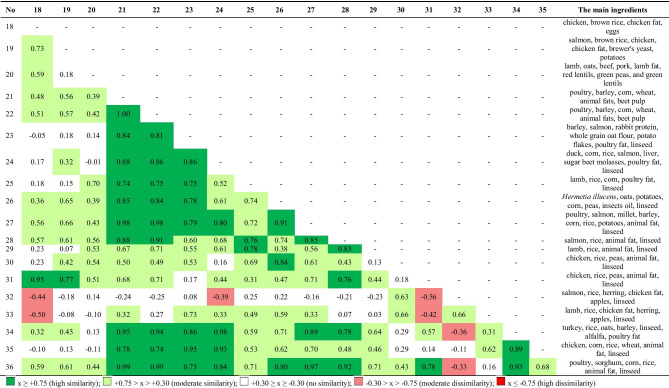
Comparative analysis of the nutritional profile (Cohen’s profile similarity coefficient) for cereal foods

**Table 4:**
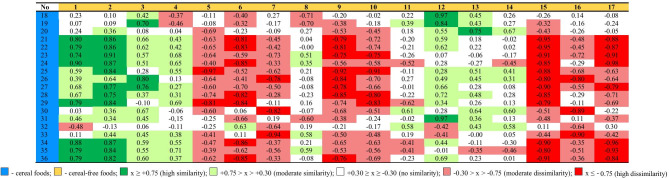
Comparative analysis of the nutritional profile (Cohen’s profile similarity coefficient) for cereal-free and cereal foods

**Table 5:** Protein: fat: carbohydrate ratio (CP: EE: NFE) and energy contribution (as energy percentage, %) of the analyzed commercial dog food

Item	CP: ME ratio	EE: ME ratio		NFE: ME ratio
1	30	28		42
2	27	24		49
3	31	29		40
4	30	21		49
5	39	33		28
6	36	44		20
7	34	38		28
8	33	29		38
9	41	38		21
10	37	37		26
11	30	39		31
12	26	32		42
13	22	38		40
14	22	40		38
15	38	41		21
16	37	36		27
17	34	48		18
18	29	34		37
19	29	29		42
20	28	35		37
21	24	20		56
22	23	21		56
23	27	16		57
24	30	17		53
25	23	30		47
26	24	24		52
27	24	23		53
28	26	27		47
29	25	28		47
30	29	32		39
31	26	31		43
32	31	32		37
33	31	23		46
34	29	20		51
35	32	22		46
36	23	20		57
Mean				
Cereal-free foods	32:35:33			
Cereal foods	27:25:48			
Dietary recommendations (Hewson-Hughes et al. 2013)			30:63:7	

*CP* crude protein, *EE* ether extract, *NFE* nitrogen free extract, *ME* metabolizable energy

**Table 6:** Microbiological analysis of the tested commercial dog food

No	TAMBC	Staphylococci (non CPS)	*Entero-bacteriaceae*	*Escherichia coli*	*Salmonella* spp.	*Listeria* spp.	*Clostridium perfringens*	*Aeromonas* spp.	TYMC
	cfu/g								
1	6.2 × 10^3^	ND	ND	ND	ND	ND	ND	ND	ND
2	> 3 × 10^7^	ND	ND	ND	ND	ND	ND	ND	ND
3	1.6 × 10^3^	ND	ND	ND	ND	ND	ND	ND	ND
4	2.5 × 10^3^	ND	ND	ND	ND	ND	ND	ND	ND
5	1.3 × 10^5^	ND	ND	ND	ND	ND	ND	ND	ND
6	5.7 × 10^5^	ND	ND	ND	ND	ND	ND	ND	ND
7	4.5 × 10^4^	ND	ND	ND	ND	ND	ND	ND	ND
8	1.4 × 10^6^	ND	ND	ND	ND	ND	ND	ND	ND
9	1.4 × 10^5^	ND	ND	ND	ND	ND	ND	ND	1 × 10^5^
10	9.6 × 10^4^	ND	ND	ND	ND	ND	ND	ND	ND
11	7.4 × 10^2^	ND	ND	ND	ND	ND	ND	ND	ND
12	1.8 × 10^4^	3.6 × 10^1^	ND	ND	ND	ND	ND	ND	ND
13	2.4 × 10^7^	5.4 × 10^2^	ND	ND	ND	ND	ND	ND	ND
14	1.8 × 10^4^	3.8 × 10^2^	ND	ND	ND	ND	ND	ND	ND
15	7.1 × 10^2^	ND	ND	ND	ND	ND	ND	ND	ND
16	8.1 × 10^3^	ND	ND	ND	ND	ND	ND	ND	ND
17	1.8 × 10^4^	8.3 × 10^2^	ND	ND	ND	ND	ND	ND	ND
18	2.2 × 10^3^	4.0 × 10^1^	ND	ND	ND	ND	ND	ND	2 × 10^2^
19	> 3 × 10^7^	ND	ND	ND	ND	ND	ND	ND	1 × 10^3^
20	9.1 × 10^2^	ND	ND	ND	ND	ND	ND	ND	ND
21	2.2 × 10^5^	ND	ND	ND	ND	ND	ND	ND	1 × 10^2^
22	2.2 × 10^5^	ND	ND	ND	ND	ND	ND	ND	ND
23	2.0 × 10^3^	ND	ND	ND	ND	ND	ND	ND	1 × 10^2^
24	1.4 × 10^3^	ND	ND	ND	ND	ND	ND	ND	ND
25	5.8 × 10^2^	ND	ND	ND	ND	ND	ND	ND	ND
26	> 3 × 10^7^	ND	ND	ND	ND	ND	ND	ND	ND
27	7.1 × 10^3^	ND	ND	ND	ND	ND	ND	ND	ND
28	3.2 × 10^2^	ND	ND	ND	ND	ND	ND	ND	ND
29	5.6 × 10^2^	ND	ND	ND	ND	ND	ND	ND	ND
30	1.4 × 10^4^	ND	ND	ND	ND	ND	ND	ND	ND
31	1.8 × 10^4^	ND	ND	ND	ND	ND	ND	ND	ND
32	8.6 × 10^2^	ND	ND	ND	ND	ND	ND	ND	ND
33	2.7 × 10^2^	ND	ND	ND	ND	ND	ND	ND	3 × 10^3^
34	1.8 × 10^3^	ND	ND	ND	ND	ND	ND	ND	ND
35	6.0 × 10^2^	ND	ND	ND	ND	ND	ND	ND	ND
36	2.7 × 10^4^	ND	ND	ND	ND	ND	ND	ND	1 × 10^3^
LS	absence	absence	3 × 10^2^ cfu/g	absent in 10 g	absent in 25 g	absence	absence	absence	˂10^4^
Ref	-	-	GMP (2005) EU (142/2011)	EU (142/2011)	GMP (2005) EU (142/2011)	-	-	-	GMP (2005)

*TAMC* total aerobic microbial count, *TYMC* total yeasts and molds count, *cfu* colony-forming units, *ND* not detected, *LS* legislative standards, *Ref* references

## Data Availability

All data generated or analyzed during this study are included in this published article.

## Abbreviations

BW: Body weight
CA: Crude ash
CF: Crude fiber
cfu: Colony-forming units
CP: Crude protein
CPS: Coagulase positive staphylococcus
DM: Dry matter
DR: Daily ration
EE: Ether extract
GMP: Good manufacturing practices
IL: Intestinal lymphangiectasia
LS: Legislative standards
ME: Metabolizable energy
MRSA: Methicillin-resistant *Staphylococcus aureus*
N: Nutrient
ND: Not detected
NFE: Nitrogen free extract
PCA: Principal component analysis
RASFF: Rapid alert system for food and feed
Ref: References
TAMBC: Total aerobic mesophilic bacteria count
TYMC: Total yeasts and molds count
